# Patient and provider perspectives on the development and resolution of prescribing cascades: a qualitative study

**DOI:** 10.1186/s12877-020-01774-7

**Published:** 2020-09-25

**Authors:** Barbara J. Farrell, Lianne Jeffs, Hannah Irving, Lisa M. McCarthy

**Affiliations:** 1 Bruyère Research Institute, 43 Bruyère St, Ottawa, Ontario K1N 5C8 Canada; 2 Department of Family Medicine, University of Ottawa, Ottawa, Ontario Canada; 3 School of Pharmacy, University of Waterloo, Waterloo, Ontario Canada; 4 Lunenfeld-Tanenbaum Research Institute Sinai Health System, Toronto, Ontario Canada; 5 Institute of Health Policy Management and Evaluation & Lawrence S. Bloomberg Faculty of Nursing, University of Toronto, Toronto, Ontario Canada; 6 Women’s College Research Institute, Women’s College Hospital, Toronto, Ontario Canada; 7 Leslie Dan Faculty of Pharmacy and Department of Family and Community Medicine, University of Toronto, Toronto, Ontario Canada

**Keywords:** Prescribing cascade, Problematic polypharmacy, Drug therapy, Older adults

## Abstract

**Background:**

Prescribing cascades occur when the side effect of a medication is treated with a second medication. The aim of the study was to understand how prescribing cascades develop and persist and to identify strategies for their identification, prevention and management.

**Method:**

This qualitative study employed semi-structured interviews to explore the existence of prescribing cascades and to gather patients', caregivers' and clinicians’ perspectives about how prescribing cascades start, persist and how they might be resolved. Participants were older adults (over age 65) at an outpatient Geriatric Day Hospital (GDH) with possible prescribing cascades (identified by a GDH team member), their caregivers, and healthcare providers. Data were analyzed using an inductive content analysis approach.

**Results:**

Fourteen participants were interviewed (eight patients, one family caregiver, one GDH pharmacist, three GDH physicians and one family physician) providing a total of 22 interviews about patient-specific cases. The complexity and contextually situated nature of prescribing cascades created challenges for all of those involved with their identification. Three themes impacted how prescribing cascades developed and persisted: varying awareness of medications and cascades; varying feelings of accountability for making decisions about medication-related care; and accessibility to an ideal environment and relevant information. Actions to prevent, identify or resolve cascades were suggested.

**Conclusion:**

Patients and healthcare providers struggled to recognize prescribing cascades and identify when they had occurred; knowledge gaps contributed to this challenge and led to inaction. Strategies that equip patients and clinicians with resources to recognize prescribing cascades and environmental and social supports that would help with their identification are needed. Current conceptualizations of cascades warrant additional refinement by considering the nuances our work raises regarding their appropriateness and directionality.

## Background

Problematic polypharmacy, defined here as using more medications than indicated or where potential harm outweighs benefit, increases people’s risk for experiencing preventable adverse events [1–3]. Prescribing cascades are a type of problematic polypharmacy [4, 5]. Classically defined, prescribing cascades occur when the side effect of a medication is interpreted as a new medical condition, resulting in a potentially unnecessary drug being prescribed to treat this new condition [6]. More recently, the notion of classifying prescribing cascades as ‘appropriate’ or ‘problematic’ has emerged [7].

To raise awareness about risks of prescribing cascades, case reports, examples and approaches to prevent, detect and reverse them have been published [8–18]. A 2018 scoping review describes education and illustrative case examples aimed at prevention and detection, approaches to using health administrative data and social media to help detect prescribing cascades and the use of medication reviews and deprescribing to reverse them [12]. Population-based observational and cohort studies examining the associations between medications that may suggest prescribing cascade phenomena have also been published and serve to inform both detection and prevention strategies [19–23]. However, no study has yet explored, from either patients’ or healthcare providers’ perspectives, the reasons why cascades occur and why they are not easily identified and reversed.

To address this gap, patients attending a Geriatric Day Hospital (GDH), their healthcare providers and caregivers were interviewed about their experiences with one or more prescribing cascades. Our study sought to gain an in-depth understanding of how prescribing cascades develop and persist, and to identify potential strategies that people feel may help identify, prevent or manage prescribing cascades. A qualitative approach was chosen to help the researchers understand and explain the perceptions and beliefs of both patients and their health care providers about this relatively new topic.

## Methods

### Design

This was a descriptive qualitative study [24–26] using one-on-one semi-structured interviews undertaken with patients of an outpatient GDH in Ottawa, Canada. In reporting the results of the current study, the checklist for qualitative studies based on the COREQ guidelines was followed [27].

### Setting

The GDH is an ambulatory program that aims to optimize the health and quality of life of frail, community-dwelling older people (aged 65 and over) referred by physicians to address two or more concerns with mobility/falls, activities of daily living, cognition, function, medication, and future planning/caregiver stress. Patients attend for half days, twice weekly, over 8–10 weeks for assessment and personalized interdisciplinary team care. This site was chosen as the setting for patient recruitment because comprehensive pharmacist-physician-led medication reviews frequently result in prescribing cascades being identified, managed and/or resolved thus providing an opportunity to identify information-rich cases [28].

### Researcher characteristics

The three investigators have a health care background, two in pharmacy (PharmD) and one in nursing (RN, PhD). The lead investigator is a clinician-researcher pharmacist who works clinically in the GDH clinic from which research subjects were identified (hereafter referred to as GDH pharmacist). She did not participate in the application of the coding schema to transcripts but did bring her perspective to the team’s interpretation of results at data review meetings allowing the research team prolonged engagement with the GDH culture. The other two investigators work as researchers at different organizations. Two research associates were also involved with one conducting the interviews and both participating in and coding the transcripts. Both research associates had masters level expertise (MA, MScN) in qualitative data collection and analysis but no experience with polypharmacy management, nor do they work clinically. All team members were female.

### Participants and sampling

**Fig. 1:**
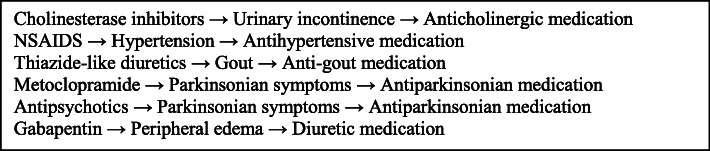
Examples of prescribing cascades

Patients who may have experienced one or more prescribing cascades were purposefully selected by the GDH pharmacist and physicians guided by, but not limited to, examples in Fig. 1: Example prescribing cascades, aiming to recruit approximately 10–15 people from both sexes and varying ages over 65, and subsequently, their caregivers and healthcare providers. Patients with severe cognitive impairment (based on GDH physician assessment) were excluded. Once several patients with similar prescribing cascades or perceptions about them had been identified, researchers asked the GDH pharmacist and physicians to identify patients with different prescribing cascades, as well as those with prescribing cascades that might traditionally be thought of as ‘appropriate,’ and those patients that might appear as outliers in terms of their experience or perspectives to ensure a broader scope of experiences was captured.

### Ethics and consent process

This study was approved by the Bruyère Research Ethics Board (protocol # M16–17-009). Physicians obtained face-to-face verbal permission from eligible patients to be contacted by the study coordinator who then obtained patients’ written consent to participate. Participants were advised that interviews were being done to understand patient, family and health care provider experiences of prescribing cascades (how they start, how appropriate they are, their impact, what challenges exist in managing them and what can be done to create ways to manage them better). Following each interview, the patient was asked for verbal permission to approach (by telephone) relevant caregivers and healthcare providers (e.g., primary care providers, GDH pharmacist and physicians) for their consent to participate in subsequent interviews about the patient’s prescribing cascade(s). If the patient agreed, caregivers and healthcare providers involved in their care were invited to participate in similar semi-structured interviews (in-person or by telephone). All participants provided written consent prior to interviews.

### Data collection

A research associate conducted all interviews (face-to-face except for one family physician interview) between May 2017 and July 2018. Interview guides were developed to focus on identifying drivers of prescribing cascades (e.g., timing of prescribing, new symptom identification, addition of the second medication); exploring circumstances when prescribing cascades might be considered appropriate; identifying behaviours that may help prevent, identify or resolve prescribing cascades; and understanding the overall clinical impact (Appendix 1) (see Additional file 1). No non-participants were present during interviews; no repeat interviews were carried out. Interviews were recorded and transcribed verbatim (files transferred in accordance with approved protocols) and transcripts de-identified and verified for accuracy against audio recordings; interviewers also recorded and saved field notes with each transcript; transcripts were not returned to participants for verification.

### Data analysis

An inductive content analysis approach was used [29, 30]. All study team members independently reviewed the first three transcripts line-by-line and then met to identify words or phrases that represented codes (January 2018). From this initial coding, a schema was developed by clustering the codes and emergent insights into categories. This coding schema was applied manually by two coders (the nursing-background co-investigator and one of the Masters level qualitative research trained staff) to the first three transcripts, then reviewed together to identify any differences, then used by each coder to analyze all of the subsequent transcripts (February 2018–August 2018). Throughout this period, ongoing coding was reviewed with the other two investigators (who were sent copies of the de-identified transcripts under discussion) at monthly teleconferences for confirmation, identification of irrelevant or erroneous coding and discussion of emerging patterns; this process provided an opportunity for the research team to be engaged in persistent observation and reflexivity. Interviews continued until data saturation was achieved when no new patterns or insights from analysis of the narrative data emerged [31]. To enhance credibility, the data set was then re-analyzed, by the same two coders using the coding schema, organized into charts with proposed themes and verbatim quotations plus impressions, then verified during a full-day in-person team retreat (September 2018) to confirm patterns, core consistencies, inconsistencies and meaning. This final analysis included developing thematic statements from the categorized data [32]. Participant checking was not conducted. Raw data (recordings, transcripts, field notes), coding schema, coded transcripts, summary products, data analysis meeting minutes (including process notes) and theme reports were filed by date to provide an audit trail.

## Results

Eight patient participants (four male, four female; age range: 70–95), one family caregiver, one GDH pharmacist, three GDH physicians and one family physician were interviewed for a total of 22 interviews about the 8 patient cases. All patients invited to participate did so; patient and caregiver interviews ranged from 30 to 45 min. Health care provider interviews ranged from 15 to 60 min. Case descriptions outlining participant characteristics including who participated in interviews about each case, the potential cascade encountered and thoughts about the cascade are depicted in Table 1 Case descriptions.

**Table 1:** Case descriptions

Case (age)	Potential cascade(s)	Sources	Interviewee thoughts about the potential cascade	Relevant quotes
1 (86)	amlodipine (ankle swelling) → furosemide (urinary frequency) → tamsulosin & dutasteride	Patient M^a^01, family physician, GDH^b^ pharmacist	Family physician thought furosemide was started prior to amlodipine and did not think furosemide was started as a result of amlodipine prescribing, assuming instead it was used for heart failure although this reason was not documented in the patient’s medical history. Patient stated that he had urinary retention when dutasteride stopped, consistent with using to manage benign prostatic hyperplasia. Little consensus as to whether a prescribing cascade had occurred.	“I would say it’s for congestive heart failure. It’s not written in the chart. But that’s my assessment because he’s in atrial fibrillation. Usually when you’re in atrial fibrillation, you’re more at risk for congestive heart failure. He came in with furosemide. I’m pretty sure it’s related to his Afib.” – Family Physician
2 (81)	diltiazem (ankle swelling) → chlorthalidone (hyperglycemia) → glyburide	Patient M02, GDH physician and pharmacist	Patient described having started diltiazem for angina, followed two weeks later by ankle swelling for which he saw his family doctor who increased his chlorthalidone dose. However his endocrinologist suggested changing diltiazem to bisoprolol, after which ankle swelling resolved and chlorthalidone was reduced to original dose then ultimately stopped due to orthostatic hypotension, following which his blood sugar fell and glyburide was reduced/stopped. Patient described that he generally monitors new drugs and reports side effects after two weeks. Prescribing cascades likely and resolved.	“…there was a heart medication, which caused my accumulation of water in my ankles, so I had to go back to the doctor… and he changed the medication because my feet were so swollen I couldn’t even put my shoes on” – Patient M02 “…the bonus was that off the chlorthalidone he noticed better blood sugars” – GDH Physician
3 (91)	amlodipine (ankle swelling) → furosemide	Patient M03, GDH physician and pharmacist	GDH physician and pharmacist stated initial plan was to reduce amlodipine, then furosemide, because blood pressure less than target with orthostatic drop and history of falls. However, these changes not done possibly because of a transient ischemic attack or small stroke while a patient at the GDH. Neither noted an ongoing reason for the furosemide except patient’s report of slight ankle edema for which he used elasticized stockings. Prescribing cascade seems likely but not resolved.	“I have taken the advice it was doing no harm and so I was told to continue it and have...” – Patient M03 “All the blood pressures are less than target, so the amlodipine could have been tapered and it just wasn’t done.” “…unknown for the furosemide. It’s possible it was started for ankle swelling for the amlodipine but I’m not 100% sure.” – GDH Pharmacist
4 (70)	prednisone (hyperglycemia) → metformin diclofenac & misoprostol (need for ulcer prophylaxis) → omeprazole	Patient F^c^04, GDH pharmacist	Patient stated prednisone started after spinal surgery seven years ago. Increasing glucose levels (not noted by anyone to be linked to prednisone). One year later, elevated glucose noted, initially controlled with diet, then metformin added one year ago. Tapered prednisone in GDH but not low enough dose to see significant reduction in glucose for potential to reduce metformin; recommended follow up after GDH discharge. Patient states omeprazole started years ago to reduce risk of ulcer with diclofenac/misoprostol then continued after it was stopped because of general polypharmacy. Prescribing cascades seem likely but not resolved.	“…she had been maintained on these for a number of years because she hadn’t had follow-up with the original prescriber in a long time.” – GDH Pharmacist
5 (71)	naproxen (need for ulcer prophylaxis) → pantoprazole → vitamin B12 naproxen (hypertension) → hydrochlorothiazide (increased uric acid) → allopurinol duloxetine & tamsulosin (headaches) → topiramate	Patient M05, GDH physician and pharmacist	Patient aware that pantoprazole useful to reduce ulcer risk with naproxen; able to reduce dose but not stop naproxen due to worsening of pain. Patient understood role for pantoprazole and also need for vitamin B12 due to reduced vitamin B12 absorption with pantoprazole. Similarly, hydrochlorothiazide dose was not reduced and patient remained on allopurinol. Reduction of duloxetine dose (in collaboration with his psychiatrist) improved his headaches and topiramate taper started. Prescribing cascades likely but difficult to fully resolve due to need for pain control with naproxen.	“…he realized that his medications could be contributing to the problem, but he didn’t know what to do next.” – GDH Pharmacist “…Naproxen is.. ideally taken for a short… time to treat inflammation and then tapered and stopped. It can cause an increased risk of GI bleeding…, high blood pressure. .. we actually decreased it .. from 500 mg.. to 250 mg but that was… the biggest stretch making that first decrease because he was really concerned about his pain significantly…” – GDH Physician
6 (88)	Quetiapine & paroxetine (tremor) → levodopa paroxetine (hypertension) → lisinopril	Patient F06, family caregiver, GDH physician	Patient and daughter stated paroxetine used for 20 years, levodopa started with Parkinson’s diagnosis about 5 years ago, then quetiapine started. They reported that no one had ever discussed that paroxetine could cause tremors or increase blood pressure. The GDH physician questioned use of levodopa as the patient had no apparent symptoms of Parkinson’s other than a previous tremor. During the GDH stay, it was noted that medication changes were difficult because of ongoing anxiety. Both paroxetine and quetiapine doses were reduced during the admission but no changes made to levodopa. Prescribing cascades difficult to verify.	“She had had a tremor at one point and possibly other signs and symptoms as well.” – GDH Pharmacist “No obvious signs of Parkinson’s Disease. I’m noting no tremor, no cogwheeling. Perhaps… subtle masked facies and her gait pattern was reasonable.. it didn’t sound like there was obvious reason for her to be on levodopa and so wondered how that came about...we never moved on the levodopa at all, and I actually never communicated with Dr. about that, which I could have done, but I never did.” – GDH Physician
7 (88)	Pregabalin & codeine & carbamazepine (confusion) → rivastigmine	Patient F07, GDH pharmacist	Patient taking several sedating medications for many years for trigeminal neuralgia; appears previous specialists recognized medication-induced cognitive impairment as likely cause for dementia. One physician communicated to patient and husband that rivastigmine unnecessary in this case but they did not want to stop it; rivastigmine subsequently increased by a different memory disorder specialist. Pharmacist noted that pregabalin was at very high dose (300 mg twice daily) possibly as a result of confusion with gabapentin. Pregabalin dose reduced greatly in GDH to lowest effective dose, with improvement in cognition, followed by reduction in rivastigmine dose. Prescribing cascade likely; partially resolved.	“I want to sleep all the time. All the time I could sleep. Always and I keep thinking it must be the medication, but people don’t listen to me.” Also “I don’t ask questions so I don’t get answers.” – Patient F07 “The patient and her husband do not feel she’d be able to function with a reduction in her pain medications and they’re willing to trade off cognitive ability.” – GDH Pharmacist
8 (95)	amlodipine (ankle swelling) → furosemide (urinary frequency)	Patient F08, GDH physician and pharmacist	Patient previously on diltiazem which worsened ankle swelling; switched to amlodipine. Patient felt ankle swelling due to previous injury, stated no-one had told her could be related to diltiazem or amlodipine. But, reluctant to reduce amlodipine because worried about blood pressure (high in the past). No changes made in GDH. Prescribing cascade likely but not resolved.	“There was extensive conversations with her about her pedal edema and her urinary symptoms and her orthostasis, and despite this, she decided not to pursue changes to her medication.” – GDH Pharmacist

^a, b, c^*Abbreviations*: *GDH* Geriatric Day Hospital, *M* Male, *F* Female

Overall, the complexity and contextually-situated nature of prescribing cascades created challenges for everyone involved with their identification. In some instances, applying a dichotomous classification where prescribing cascades are deemed ‘problematic’ or ‘appropriate’ was too polarizing because the clinical circumstances changed over time. For example, one study participant was taking a non-steroidal anti-inflammatory drug (NSAID), naproxen, for pain relief but was unable to confirm its’ clinical benefit. To reduce the risk of stomach ulcers (a potential adverse effect of NSAIDS), the patient was also taking a proton pump inhibitor (PPI), pantoprazole. In this situation, the prescribing cascade of NSAID and PPI appear ‘problematic’ because the ongoing benefit of the NSAID was unknown. The dose of the NSAID was lowered with the aim to taper toward cessation and hope that the PPI could be discontinued to resolve the cascade. However, with the lowered dose of the NSAID, the patient reported increased pain so the decision was made to continue the medication combination thereby rendering the prescribing cascade ‘appropriate’. Interviewees also spoke about situations where a patient might have eventually developed a condition that required treatment regardless of a medication precipitating it. For example, in the situation where a patient was taking prednisone, then prescribed metformin, would the patient have developed diabetes (and required metformin) regardless of taking the prednisone? Another study participant had an increase in dose of an existing medication used to treat dementia (rivastigmine) following an increase in dose of a concurrent pain medication (pregabalin) that can cause confusion. In this case, the dose of pregabalin was reduced, confusion improved and then the rivastigmine dose was reduced. This example led the team to discuss the concept of ‘directionality’ of assessing a prescribing cascade (i.e., the patient is already taking a medication that requires a dose increase following the addition or dose increase of another medication). Such complexity in these cases contributed to difficulty in reaching consensus about whether a prescribing cascade was indeed occurring and how it should be managed.

### Emergent themes

Three interrelated themes emerged as we explored how prescribing cascades develop and persist: varying awareness of medications and cascades; varying feelings of accountability for making decisions about medication-related care; and accessibility to an ideal environment and relevant information. Potential actions to prevent, identify or resolve cascades were also proposed.

#### Varying awareness of medications and cascades

Patients and their healthcare providers expressed varying degrees of awareness about reasons for, and experience with the medications taken by patients. Few patients or providers recognized that a prescribing cascade may have occurred; no patients were aware of the term ‘prescribing cascade’. Some patients had basic knowledge about the medications they take, e.g., able to recall names and reasons for use. Many could not recall when medications were started or the order in which they were prescribed. Even fewer recognized symptoms experienced as potential side effects of existing medications. Some felt that their understanding of medications was negatively impacted by having had changes made during times of stress e.g., during hospitalizations: *“I was really upset when I was in the hospital, I didn’t remember really what the doctor told me about everything”* (M01). Some reported having been well-versed in their medications and reasons for their use in the past but sensed they had, “*lost track*” (F07) of their medications as their health declined or number of medications increased: *“I just take whatever I take and I don’t worry about it now. I know that sounds pretty terrible. It wasn’t always this way”* (F07).

Few patients could articulate specific goals of care related to their medications. Some lacked awareness of usual durations for medication use and potential non-pharmacologic management strategies for their conditions. Patients who did have basic knowledge about their own medications typically had completed more formal education or undertook self-study to gather this information. One patient, who had completed significant formal education, described his proactive approach to managing his own medications: “*I am the medium*” (M02). This person described multiple strategies he used to acquire information about medications and their adverse effects and was an outlier in terms of monitoring for adverse effects and reporting them to the family physician*: “My thinking was, if this is causing this accumulation of water in the ankles, it is not desirable so I made an appointment and he agreed”* (M02).

For physicians, awareness largely refers to whether they recognized that a symptom was related to a current medication. In some cases, physicians recognized an adverse effect but opted to prescribe a second drug rather than make a change to the first medication. The gradual onset of adverse effects sometimes made recognizing a cascade hard because a clear temporal relationship was lacking. In other cases, physicians described uncertainty about what constituted a prescribing cascade: *“I don’t believe that’s a prescribing cascade, but what we see is NSAIDs followed by PPIs for stomach protection”* (family physician for M01). Sometimes physicians incorrectly identified cascades as being appropriate when they might not have been: *“with prednisone, we use a PPI to protect the stomach”* (family physician for M01).

Physicians reported that they used patients as information sources about medication experiences but confirmed that the depth of information provided varies amongst patients. Amongst patients who could identify a reason for use, many still struggled to provide the chronology of their medications. Patients with cognitive or intellectual challenges were less likely to be knowledgeable about their medication use. When there was a reliable historian (patient or caregiver) to supply this information, it was, *“invaluable in helping out with the process of discovering a prescribing cascade and recognizing it”* (GDH physician for M02 and F06). Physicians often tried to triangulate information sources to compile a history of medication use and effects, but the process was time-consuming, so they relied largely on efforts of team members, especially the pharmacist.

Patients found attempts to gain medication knowledge from, or to ask questions of, prescribers challenging. Some felt unheard and shared having given up on trying to engage them in discussion: *“I keep thinking it must be the medication, but people won’t listen to me… Maybe because I don’t ask the questions, I don’t get the answers”* (F07). This may be aggravated by short appointments, which leave patients feeling rushed and sensing that clinicians prescribed without worrying about side effects.

#### Varying feelings about accountability for making decisions about medication-related care

Healthcare providers and patients experienced varying levels of accountability (i.e., responsibility or authority) for participating in decisions about medication-related care. Healthcare providers consistently felt accountable for decisions they made: *“I think it’s my job to check the medication every time the patient comes in”* (family physician for M01) but did not always take action, particularly if they lacked information or there was patient resistance to change. Patients viewed their accountability more variably, with a few having a stronger sense of accountability than others and different preferences for roles in decision-making. Specifically, patients’ interests in participation in their care ranged from none to a firm belief that they are a partner in care - responsible for communicating their experience and needs. Those with less interest in participation had trouble asking questions or were content to take direction from a healthcare provider. Others described their roles as communicators and advocates for their health, eager to participate in decision-making: *“The patient has to communicate and be forthcoming, otherwise, how can they make decisions, for example family doctors, I am not the only one, she has hundreds of them, so unless I tell her, how will she know?”* (M02).

Healthcare providers reported that they were accountable for communication amongst themselves and with patients but that better information would help identify, prevent or resolve prescribing cascades. Both physicians and pharmacists believed the responsibility for educating patients, particularly reviewing side effects, was shared and important because this may facilitate identifying side effects that, unrecognized, could result in prescribing cascades. Both felt that when side effects or prescribing cascades were identified, they were responsible for including patients in exploring options, priorities, shared goals and decision-making.

Ultimately, our team interpreted these findings surrounding accountability in the context of prescribing cascades in the following ways: healthcare providers could make better decisions and share responsibility for medication-related care if patients asked them about specific symptoms, were forthcoming about their conditions and participated in making decisions about plans for medication-related care; the best care requires awareness by patients, caregivers and healthcare providers about the reasons medications are prescribed and their intended effects.

#### Accessibility to an ideal environment and relevant information

Practice environment and system issues support and constrain identifying and resolving prescribing cascades. The GDH team and structure (including a pharmacist who coordinated dosage changes and monitoring) provided an environment conducive to identifying and resolving prescribing cascades. Having both the prescriber and the pharmacist engage in discussions with patients to set shared goals of care, goals for each medication, review risks and side effects of each medication and the potential impact of stopping medications facilitated patient buy-in for deprescribing activities to resolve cascades. The discussions led to shared decision-making about options for tapering (or not) and ultimately impacted the ability to make and monitor medication changes. Frequent visits allowed for both nimble changes and reassurance for patients who could be initially reluctant to make changes:

*“One of the keys to success is that we tell people, we’re going to see them again in two days, so people are not feeling as though they’re going to be left suffering or struggling with a symptom for weeks without being able to access care and support and a way to modify the regimen again if it’s not working.” (*GDH physician for patient M05*)*

Access to patients’ charts allowed team members to share information like vitals, lab test results and care plans. Communication using progress notes, medication orders and prescriptions allowed the team to share the rationale for medication changes, monitoring and next steps. Sharing this information through transitions in care (e.g., on prescriptions transferred to a community pharmacist and family physician) allowed for consistent reinforcement of important messages:

*“When we write our prescriptions, we always write the indication and if there’s a change in the dose or if it’s being stopped altogether, we’ll write the reason why that’s happening…and the prescription gets faxed to the family physician’s office and to the community pharmacy. So the hope is that if we’re better at letting each other in on our thought process, then everybody can reinforce the same message.”* (GDH physician for M05)

Despite this optimal environment for identifying and resolving prescribing cascades, constraints existed. When the pharmacist was not present to screen patients for possible prescribing cascades and study eligibility, the physicians found it difficult to do so on their own. This was partly due to lack of awareness of potential prescribing cascades and a thought process learned in medical school which focused on treating symptoms rather than assessing whether symptoms could be drug-induced.

*“When you look at medical training, when somebody comes in with a symptom, we learn a differential diagnosis for what the possible causes are, and if you’re writing a test in medical school, you can always put drugs on your differential for anything that somebody comes with, but it’s kind of a very theoretical thing. It never really gets talked about more specifically than that.”* (GDH physician for M05)

The GDH health care providers also identified historical information gaps (patient-specific knowledge about reasons for medication use and effects, rationale from specialists or hospital admissions for prior medications) as a barrier to identifying prescribing cascades. In most cases, there was low consensus that a prescribing cascade had occurred, often due to this lack of information and conflicting reports about indications and chronology.

Physicians also described competing care demands throughout the GDH admissions which meant medication recommendations to address prescribing cascades were summarized in discharge reports rather than being operationalized.

### Actions that could help

Interviewees shared possible interventions for preventing, identifying and resolving prescribing cascades (Table 2). These involved role changes (e.g., for pharmacists and nurse practitioners); others encompassed behavioral changes (e.g., having prescibers document reasons for medications) and others included workflow changes or educational activities.

**Table 2:** Potential actions to help prevent, detect or resolve prescribing cascades

1	Increase the role for community pharmacists and for pharmacists or nurse practitioners working in interprofessional primary care teams, to conduct medication education and review, including the potential for using medical directives to facilitate monitoring and prescribing
2	Have prescribers document the reason for medication use, and rationale for changes, on each prescription
3	Facilitate access to complete and centralized electronic health records by all members of the health care team (to document, provide and share information about medication use, chronology and experience)
4	Apply judicious use of alerts in electronic health records, pharmacy dispensing systems and medication reviews for medication combinations that could be prescribing cascades
5	Confirm the existence of a prescribing cascade through deprescribing and monitoring
6	Modify primary care and pharmacy workflow to increase frequency of follow-up when medication changes are made for older people who may lack awareness or capacity to monitor the effects of medications
7	Incorporate prescribing pitfalls (including prescribing cascades) with tangible examples into medical education
8	Educate the public encouraging them to ask questions about their medications
9	Provide patient education (in lay language and sensitive to cultural context and varying cognitive abilities) about medication purposes and side effects to look for

## Discussion

To our knowledge, this is the first qualitative exploration of patient and provider perspectives regarding prescribing cascades. We found that gaps in knowledge about the purpose, chronology and effect of medications hamper patients’ abilities to communicate with their healthcare providers in ways that allow for effective monitoring and interpretation of their effects. Healthcare providers similarly often lack this information, limiting their ability to recognize when a prescribing cascade is possible. Despite feeling accountable for medication-related care, healthcare providers may not act when they lack information or do not have access to a conducive environment.

In addition to exploring patients', caregivers' and healthcare providers’ perspectives about cascades broadly, we originally sought to understand how prescribing cascades develop and persist. Further, we aimed to identify strategies for practical identification, prevention or management of cascades. Yet early in our interviews and analysis we recognized a key challenge is that patients and healthcare providers often do not recognize when prescribing cascades have occurred. This led us to narrow our focus toward elucidating opportunities to further refine the concept of cascades and describe factors that influence their recognition and resolution where possible.

The classical definition of the term ‘prescribing cascade’ has been refined to encompass the concepts of appropriate or problematic; intentional or unintentional; and recognized or unrecognized prescribing cascades [7]. Our findings suggest further nuances of these concepts are worthy of consideration with dichotomies being too polarizing. For example, prescribing cascades can move from being problematic to appropriate when managed by interventions like dose reduction. As well, circumstances involving the worsening of a present condition with the addition of another medication or the improvement of a condition with the addition of a medication (resulting in deprescribing a medication used to treat the condition) need to be considered. This example suggests the need for a discussion of the directionality of a prescribing cascade as well as the impact of a prescribing cascade on drug interactions affecting control of another disease or symptom.

This complexity contributes to the challenges in recognizing and reaching consensus about when a prescribing cascade has occurred. Recognizing and confirming the presence of adverse drug reactions led to the development of the Naranjo scale [33]. Our findings support the need to develop a similar scale that identifies and confirms the existence of a prescribing cascade. Preliminary work with a small number of patient cases to draft a scoring system for the likelihood and severity of a prescribing cascade has been published and deserves attention, taking into account the perspectives emerging from our study [13]. Further, some have recommended developing alerts for electronic health records and pharmacy dispensing systems that identify particular medication combinations that may represent a cascade, an intervention which is likely to be welcomed by pharmacists [34, 35]. At least one pharmacy school has taken steps to incorporate the concept and examples of prescribing cascades into its curriculum through a game-based recognition activity [36]. This approach could be explored further across the pharmacy, medicine and nursing curricula. Without better ways for health care providers to recognize prescribing cascades, efforts to prevent and resolve them may be futile.

To effectively estimate the probability that a cascade has occurred, one needs to know, at a minimum, the reasons a medication is prescribed and when it was started in relation to other medications. Concerns over lack of indication for prescribed medications that older people are taking have been demonstrated elsewhere [37]. We found healthcare providers, unable to access clinical information systems with this data, typically relied on patients and families to provide this information; however, patients and caregivers could not reliably provide it. Once prescribing cascades are more easily recognized, further work can explore perspectives about their appropriateness and clinical impact as well as what people do with this new knowledge.

Our findings are drawn from patients receiving care in one setting with primarily the GDH pharmacist and physicians as healthcare providers; only one family physician was included. As such, the transferability of our findings to other settings (e.g., primary care, other day programs, hospitals and nursing homes) may be limited. Mild cognitive limitations may have also affected patients’ abilities to contribute to the story of their potential prescribing cascade. While the intention was to interview patient caregivers, only one agreed to participate; given that family caregivers may have knowledge about the patient’s medication experience, the relative lack of this perspective is an important limitation.

Despite these limitations, our study is important because it is the first to explore patients and healthcare providers’ perspectives, highlighting the struggles encountered with applying the concept of prescribing cascades and recognizing them in practice. Our research team is continuing this study, focusing on exploring the concepts of appropriate versus problematic cascades and expanding patient recruitment in both community and long-term care settings. A self-referral stream will allow recruitment of people who may have more knowledge and feelings of accountability.

## Conclusion

We found that patients’ and healthcare providers struggled to recognize prescribing cascades and identify when they had occurred. Gaps in knowledge about the reasons for use, when a medication was prescribed, and its expected benefits abound. This impacts patients’ abilities to partner with healthcare providers to identify or resolve prescribing cascades. Further, clinicians’ struggles with similar knowledge gaps may lead to inaction toward resolving the cascade, despite feeling accountable for medication-related care.

Our study has several implications, highlighting the need for strategies that equip patients and clinicians with resources to recognize prescribing cascades, environmental and social supports that would help with their identification, and suggesting that current conceptualizations of cascades warrant additional refinement by considering the nuances our work raises.

## Supplementary information

**Additional file 1.** Interview guides. The guide used to interview participants.

## Data Availability

The interview transcripts generated and analyzed during the current study are not publically available as participants consented only to the use of their de-identified material by investigators who had signed confidentiality forms, and relevant Research Ethics Boards audit committees. Summary datasets generated during the analysis phase are available from the corresponding author on reasonable request.

## Abbreviations

COREQ: Consolidated criteria for reporting qualitative research
GDH: Geriatric Day Hospital
NSAID: Nonsteroidal anti-inflammatory drugs
PPI: Proton pump inhibitor
M: Male
F: Female
