# Supplementary material for: Patient and provider perspectives on the development and resolution of prescribing cascades: a qualitative study
Source: BMC Geriatr. 2020 Sep 25;20:368. doi: 10.1186/s12877-020-01774-7 (PMC7519478; doi:10.1186/s12877-020-01774-7)
Supplement: Supplementary file 1 — Additional file 1. Interview guides. The guide used to interview participants. [file 12877_2020_1774_MOESM1_ESM.docx]

**Appendix 1:** Interview guides

**Understanding Prescribing Cascades: Patient Interview Guide**

**Note:** Once the patient has consented to participate, specific medication names and related symptoms will be supplied by either the GDH pharmacist or physician to the research assistant to fill in the blanks in the interview guide and to facilitate the interview process.

**Preamble**

Thank you for agreeing to participate in this study. My name is <insert name> and I am a research assistant with the Institute. Our study team is hoping to hear about your experiences with your medications, more specifically about the time when you were given a medication to manage a side effect from another of your medications. I am going to ask you a series of questions but want to assure you that there are no right or wrong answers. We are interested in your thoughts and experiences. The interview will last about 30 to 45 minutes but if at any time you would like a break or want to stop and continue at another time, let me know and we can do that.

I’m going to be taking notes but that’s just to help my memory.

**Icebreaker**

1. First off, I’d like to get to know a little more about you and your medication experiences.
2. How old are you?
3. What is your highest level of education?
4. How did you end up going to the Day hospital?
5. Can you tell me how you feel in general about the medications you’re taking?

**Probes:**

1. How do you feel about the number/types of medications you take? (e.g. too many, just right, need more?)
2. How do you like to learn about your medications-who usually does the teaching?
3. Have you ever had questions about your medications? How did you get them answered?
4. How do you feel about the medication change you had while at the Day hospital?

**Probes:**

1. What was that process like?
2. Did you learn anything new about your medications while you were there?
3. How have the changes gone? (e.g. process, impact?)

**First Prescribing Cascade**

**Focus on Medication X**

We’re now going to focus on the prescribing cascade that has been identified. As a reminder, a prescribing cascade happens when a new medication is started to help you manage a side effect that is caused by one of your other medications. Sometimes these are very appropriate but other times they might have been prevented if the first medication had been recognized as the cause of the side effect and its dose reduced or stopped.

1. Thinking back, when did medication x <use name of medication> start? Note: If the participant cannot remember exactly, probe for general time frame i.e., within the last year, 5, or 10 years.

**Probes:**

1. Indication for use
2. Identity of original prescriber
3. Relative to that, when did <insert patient’s name> start to experience the <use name of symptom(s)> that bothered her/him?
4. Tell me more about what the <use name of symptom(s)> were like and what you did about it/them.

**Probes:**

1. How did the symptoms present?
2. What actions did the patient take?

At some point, <insert patient’s name> spoke to the person who prescribed that medication for her/him about the symptoms you had.

1. About how much time had passed taking medication x before <insert patient’s name> had this discussion?
2. What did the person who prescribed it say at that time about their impression of the situation?

**Probes:**

1. What was the patient told?
2. Whether a discussion about potential causes (i.e., drug side effect) occurred
3. What options were discussed for management?

**Focus on Medication Y**

After that discussion with your prescriber, you received a prescription for a new medication, called y, to help with the symptoms.

1. Thinking back, what did you do next?

**Probe:**

1. How long did you wait before you started to take this new medication?

1. How did the new medication help (or not help) with the symptoms that were bothering you?
2. Was there anything in particular that you remember doing to keep track of whether it helped? If so, can you tell me more.

**Probes:**

1. How about your prescriber, did they talk to you about side effects?
2. Did you see them or speak with them more often after the medication change?
3. What did they do to keep track of whether the new medication helped?
4. At the time, did you notice changes to your:

A. balance,

B. memory,

C. overall ability to do the things you normally did?

**Probe:**

1. For each of A., B., C., what changes did you notice?

**Medications X and Y**

1. About how long did you take both medications X and Y together?

**Probe:**

1. Still taking the combination?

1. What happened to make it come to light that that the symptoms you first had were likely related to medication X?

**Probes:**

1. Who identified it?

2. How was the issue managed or resolved?

3. How did learning about the possible prescribing cascade make you feel?

1. Once either X or Y, or the combination of X and Y was stopped, what do you observe with respect to your original symptoms? And what did you observe about your balance, memory, ability to do things you normally do (ask one at a time)?

**Recommendations**

1. Thinking back, what things might have helped uncover the situation of having the side effect of a drug treated by another drug earlier or maybe have prevented it?

**Probe:**

1. Things patient, prescriber, pharmacist or other health professional could have done (e.g. medication review; asking if a new symptom could have been caused by a drug; list of side effects to watch for when new medication is prescribed, for example, at hospital discharge)

1. What things do you think people need to do differently to prevent, manage or resolve prescribing cascades?

**Probe:**

1. Behaviours, suggestions for others i.e., people, health professionals (e.g., medication reviews, electronic health records)

**Conclusion**

Thank you for your time, and for your contribution to our research study.

We are looking for additional perspectives on the prescribing cascade that you experienced. If you have a family member who provides care for you who you feel would be suitable to provide another outlook on this prescribing cascade, would you like them to participate in this study?

[If yes, give patient a copy of Study Invitation Letter for Family Caregivers (Appendix G) to give to their family member]

[If no, continue to next prompt]

Would you like to provide us with contact information for a member of your health care team who you feel would be well-suited to provide us with more perspectives on the prescribing cascade you experienced? This person can be your family doctor, a specialist, your pharmacist, nurse, or other health care worker.

[If yes, record the health care provider’s contact information]

[If no, thank the patient again and leave]

Health care provider’s full name, contact number or email: ____________________________________

**Understanding Prescribing Cascades: Family Caregiver Interview Guide**

**Note:** Once the patient and caregiver has consented to participate, specific medication names and related symptoms will be supplied by either the GDH pharmacist or physician to the research assistant to fill in the blanks in the interview guide and to facilitate the interview process.

**Preamble**

Thank you for agreeing to participate in this study. My name is <insert name> and I am a research assistant with the Institute. Our study team is hoping to hear about <insert patient’s name>’s experiences with her/his medications, more specifically about the time when <insert patient’s name> was given a medication to manage a side effect from another of their medications. I am going to ask you a series of questions but want to assure you that there are no right or wrong answers. We are interested in your thoughts and experiences. The interview will last about 30 to 45 minutes but if at any time you would like a break or want to stop and continue at another time, let me know and we can do that.

**Icebreaker**

1. First off, I’d like to get to know a little more about <insert patient’s name> and her/his medication experiences.
2. Can you tell me how you feel in general about <insert patient’s name>’s medications and any changes s/he had while s/he has been at the Day Hospital?

**Probes:**

1. How do you feel about the number/types of medications <insert patient’s name> takes? (e.g. too many, just right, need more?)
2. How have changes gone? (e.g. process, impact?)

**First Prescribing Cascade**

**Focus on Medication X**

We’re now going to focus on the prescribing cascade that has been identified. As a reminder, a prescribing cascade happens when a new medication is started to help a patient manage a side effect that is caused by one of their other medications. Sometimes these are very appropriate but other times they might have been prevented if the first medication had been recognized as the cause of the side effect and its dose reduced or stopped.

For <insert patient’s name>, we are specifically interested in what led to <insert patient’s name> taking medications x <insert name of medication 1> and y <insert name of medication 2>.

1. Thinking back, when did medication x <use name of medication> start? Note: If the participant cannot remember exactly, probe for general time frame i.e., within the last year, 5, or 10 years.

**Probes:**

1. Indication for use
2. Identity of original prescriber
3. Relative to that, when did <insert patient’s name> start to experience the <use name of symptom(s)> that bothered her/him?
4. Tell me more about what the <use name of symptom(s)> were like and what you did about it/them.

**Probes:**

1. How did the symptoms present?
2. What actions did the patient take?

At some point, <insert patient’s name> spoke to the person who prescribed that medication for her/him about the symptoms you had.

1. About how much time had passed taking medication x before <insert patient’s name> had this discussion?
2. What did the person who prescribed it say at that time about their impression of the situation?

**Probes:**

1. What was the patient told?
2. Whether a discussion about potential causes (i.e., drug side effect) occurred
3. What options were discussed for management?

**Focus on Medication Y**

After that discussion with <insert patient’s name>’s prescriber, s/he received a prescription for a new medication, called y, to help with the symptoms.

- 1. Thinking back, what did <insert patient’s name> do next?

**Probes:**

1. How long did the patient wait before s/he started to take this new medication?

1. How did the new medication help (or not help) with the symptoms that were bothering <insert patient’s name>?
2. Was there anything in particular that you remember <insert patient’s name> doing to keep track of whether it helped? If so, can you tell me more.

**Probe:**

1. How about <insert patient’s name> prescriber, what did they do to keep track of whether the new medication helped?
2. At the time, did you notice changes to <insert patient’s name>’s:

A. balance,

B. memory,

C. overall ability to do the things s/he normally did?

**Probe:**

1. For each of A., B., C., what changes did you notice?

**Medications X and Y**

1. About how long did <insert patient’s name> take both medications X and Y together?

**Probe:**

1. Still taking the combination?

1. What happened to make it come to light that that the symptoms <insert patient’s name> first had were likely related to medication X?

**Probe:**

1. Who identified it, how was the issue managed or resolved?

1. Once either X or Y, or the combination of X and Y was stopped, what do you observe with respect to <insert patient’s name>’s original symptoms? And what did you observe about <insert patient’s name>’s balance, memory, ability to do things s/he normally does (ask one at a time)?

**Recommendations**

1. Thinking back, what things might have helped uncover the situation of having the side effect of a drug treated by another drug earlier or maybe have prevented it?

**Probes:**

1. Things patient, prescriber, pharmacist or other health professional could have done (e.g. medication review; asking if a new symptom could have been caused by a drug; list of side effects to watch for when new medication is prescribed, for example, at hospital discharge)

1. What things do you think people need to do differently to prevent, manage or resolve prescribing cascades?

**Probe:**

1. Behaviours, suggestions for others i.e., people, health professionals (e.g., medication reviews, electronic health records)

**Conclusion**

Thank you for your time, and for your contribution to our research study. Your input is greatly appreciated!

**Understanding Prescribing Cascades: Health Care Provider Interview Guide**

**Note:** For the purposes of this guide, health care providers invited to participate are one of the following people who were identified by a patient or caregiver as being able to speak about the patient’s experiences with prescribing cascades: family doctor, a specialist, pharmacist, nurse, or other health care worker. Herein, they will be called “health care providers”

Once the health care provider has consented to participate, specific medication names and related symptoms will be supplied by either the GDH pharmacist or physician to the research assistant to fill in the blanks in the interview guide and to facilitate the interview process.

We recognize that there seem to be more questions than are possible to answer within a 15-30 minute time period. We anticipate that most health care providers will not be able to recall when or how prescribing cascades evolved so some questions will result in very short answers. However, we’ve been inclusive of all possible questions. If the interview reaches 30 minutes, the interviewer will explain that the allotted time is complete and ask permission to continue (anticipating that this may not be possible in some situations).

**Preamble**

Thank you for agreeing to participate in this study. My name is <insert name> and I am a research assistant with the Institute. Our team is grateful to you for taking time from your schedule today to share with us your experiences about managing pharmacotherapy for older adults in general and with prescribing cascades more specifically. As a reminder, a prescribing cascade occurs when the side effect from a medication leads to a second drug being started to manage the side effect.

You were invited to participate in this study because <patient name> or a caregiver identified you as someone able to speak to their experience with one or more prescribing cascades.

Before we get started, the plan today is to pose a series of questions to gather your thoughts and experiences. Rest assured that we are not looking for a particular answer and that nothing will be viewed as correct or incorrect in your responses. The more you are willing to share will help our team as a longer-term goal is to build tools, strategies or interventions that can assist with identifying, preventing or managing prescribing cascades. The interview will last about 20 to 30 minutes but if at any time you would like a break or want to stop and continue at another time, let me know and we will make arrangements.

**Icebreaker**

1. To get started, can you tell me about what you have seen with regard to prescribing cascades in general?

**Probes:**

1. Any specific types of cascades commonly seen?

**First Prescribing Cascade**

**Focus on Medication X**

We’re now going to focus on (one of) the prescribing cascades that has been identified in your patient. By prescribing cascade we mean, a time when the side effect from a medication leads to a second drug being started to manage the side effect. We also want to acknowledge that sometimes prescribing cascades are inevitable as they can be a trade-off of risks and benefits for patients. However, in other situations, these may have been prevented if the first medication had been recognized as the cause of the side effect and its dose reduced or stopped.

For <patient name>, we are specifically interested in what led to <patient name> being prescribed medications x <insert name of medication 1> and y <insert name of medication 2>.

1. Thinking back, when was <patient name> first prescribed medication x <use name of medication>? Note: If timing uncertain, probe for general time frame i.e., within the last year, 5, or 10 years.

**Probes:**

1. Indication for use
2. Identity of original prescriber

If interviewee not original prescriber:

1. Relative to that, at what point did you become involved in <patient name>’s care? or Has the original prescriber followed the patient with respect to monitoring impact of the medication, or have you been doing that? (question depends on whether the interviewee is a prescriber or not)

**Probes:**

1. Indication for use

2. Identity of original prescriber

1. Back to the prescribing cascade, what is your understanding about when <patient name> started to experience side effects related to the medication?

**Probes:**

1. How did the symptoms present?

2. What actions did the patient take?

1. At some point, <patient> spoke to a health care provider about these symptoms or the provider became aware of them. How did this happen?

**Probes:**

1. Identity of who patient spoke to i.e., interviewee or another provider, circumstance

1. Relative to this first reporting of the side effect, at what point did you become involved in in <patient name>’s situation with medication x?

**Probe:**

1. Role in identifying and/or managing the cascade

1. What do you recall about how the cascade was managed?

**Probe:**

1. What patient was told, whether patient was informed about potential causes (i.e., the cascade), what management options were considered

**Focus on Medication Y**

We’re now going to turn our discussion to the medication that was prescribed to manage the side effect.

At some point, <patient name> was prescribed a new medication, called y, to manage the symptom (or side effect) being experienced.

1. What do you recall about the discussion that occurred between health providers (or if prescriber, your thought process) at that time?

**Probes:**

1. Other management options considered, rationale for prescribing medication Y

2. Was the idea that this could be a prescribing cascade considered or discussed?

1. How did the new medication help (or not help) with the symptoms that were bothering <insert patient’s name>?
2. Once the second medication was started, what was the response?

**Probe:**

1. Report back if specific symptoms occurred, report back in particular time frame

1. At the time that all of this was happening, how was <patient name> doing with respect to his/her overall function?

**Probe:**

1. Balance, memory, ability to perform activities of daily living

**Deprescribing Medications X and/or Y**

Next, we’d like you to think back to when the symptom that Y was being used for was identified as possibly being caused by X.

1. What happened to make it come to light that the symptoms being experienced were a side effect related to medication X?

**Probe:**

1. Who identified it?

2. How was the issue managed or resolved?

1. Once either X or Y, or the combination of X and Y was stopped, what happened with <patient name>?
2. Did you observe any changes with respect to overall functioning?

**Probe:**

1. Balance, memory, ability to perform activities of daily living

**Recommendations**

Now we would you like you to reflect on things that can be done in daily practice to improve the prevention, detection and management of prescribing cascades.

1. Can you think of anything that could have been put in place from your perspective as a provider that may have helped detect the cascade earlier or prevent it entirely? How about from a patient perspective?

1. Were there systems or structures or processes in place at the time or that are now in place that you think might help identify, prevent or resolve prescribing cascades?

**Probe:**

1. examples including medication review; asking if a new symptom could have been caused by a drug; list of side effects to watch for when new medication is prescribed, for example, at hospital discharge

1. At the individual patient-provider level, what do you think needs to change to increase awareness about prescribing cascades with the goal of preventing, managing, or resolving them?
2. At a practice level, what strategies can you think of, they don’t necessarily have to be in place now, that could be put in place to help prevent, identify, manage or resolve prescribing cascades?
3. At a health system level, what strategies can you think of, they don’t necessarily have to be in place now, that could be put in place to help prevent, identify, manage or resolve prescribing cascades?

**Probe:**

1. If participant is uncertain, offer one example at a time. Examples might be scheduled medication reviews at particular intervals or alerts built into e-prescribing systems or electronic health records.

**Conclusion**

Thank you very much for your time. It’s been much appreciated. We’ll be collating results from all of our interviews and would be happy to let you know when the final overall results are available.
